# Correlation Between Urodynamic and Clinical Diagnoses in Classifying the Type of Urinary Incontinence in Women

**DOI:** 10.7759/cureus.6016

**Published:** 2019-10-28

**Authors:** Konstantinos Palaiologos, Manjula Annappa, Georgios Grigoriadis

**Affiliations:** 1 Obstetrics and Gynecology, Diana Princess of Wales Hospital, Grimsby, GBR; 2 Obstetrics and Gynaecology, Diana Princess of Wales Hospital, Grimsby, GBR; 3 Obstetrics and Gynaecology, Hull Royal Infirmary, Hull, GBR

**Keywords:** urodynamic investigations, urinary incontinence, diagnostic test

## Abstract

Urodynamic tests are considered the gold standard for investigating and managing patients with urinary incontinence symptoms. The objective of this review is to determine the correlation between urodynamic and clinical diagnoses in identifying the type of urinary incontinence in pre and postmenopausal women. This is a retrospective review of 116 women with urinary incontinence symptoms that were evaluated clinically initially and then investigated further with urodynamic tests. The results of the urodynamic studies were compared with the diagnosis assigned based on the clinical evaluation. For 69 women, the clinical diagnosis was not confirmed by urodynamic tests. In addition to this, the clinical diagnosis was confirmed by urodynamic investigations in only 38% of the patients. This is in accordance with current literature, which is suggestive that the agreement between urodynamic studies and clinical evaluation in identifying the type of urinary incontinence is poor. Larger definite trials are needed to provide further evidence of the diagnostic value of urodynamic tests in the management of patients with urinary incontinence symptoms.

## Introduction

Urinary incontinence is defined as involuntary urine loss and the inability to retain urine in the bladder between voluntary acts of urination [1]. It often causes social and hygiene problems. Based on the International Continence Society (ICS) in 2002, urinary incontinence is divided into three main types based on the symptoms, which are stress urinary incontinence, urge urinary incontinence, and mixed urinary incontinence [2].

Urodynamic investigations are a functional assessment of the lower urinary tract, and they are widely considered the gold standard in the assessment and management of women with urinary incontinence. The term includes numerous physiological tests of bladder and urethra function that aim to diagnose an underlying abnormality of either storage or voiding [3]. The Royal College of Obstetrics and Gynaecology recommends urodynamic testing prior to surgery for stress incontinence for patients with suspected complicating factors such as voiding dysfunction or detrusor overactivity [4]. In spite of this, there is not enough evidence to suggest that their use improves clinical outcomes [5]. It has also been shown that there may be a poor correlation between the type of urinary incontinence based on clinical evaluation and the results of urodynamic investigations [6-7].

## Materials and methods

This is a retrospective review of 116 women who were seen in the Urogynaecology Clinic and had urodynamic investigations for urinary incontinence between May 2014 and May 2016 in Diana Princess of Wales Hospital, Grimsby, UK. We collected the medical notes of each case and reviewed the clinical diagnosis based on each patient's symptoms and medical history at the first clinic appointment. Then, we ascertained the result from the urodynamic investigations that were performed following that appointment by either checking the medical notes or accessing the medical records through the hospital's health software. Following this, we determined at what percentage of each type of urinary incontinence based on clinical evaluation (stress urinary incontinence, overactive bladder, and mixed urinary incontinence) was confirmed by the results of urodynamic investigations. We also included data on ethnicity, age, menopausal status, body mass index (BMI), parity, previous mode of delivery, previous gynecological procedures, relevant comorbidities, evidence of pelvic organ prolapse, and associated symptoms.

## Results

One-hundred sixteen patients were included in our study. The main ethnicity was White British (98%). Seventy-six patients (65%) were post-menopausal and 51 patients (44%) had BMI>30. One hundred and nine patients (94%) were multiparous, of which 7% had previous instrumental delivery and 7% had a previous cesarean section. Seventy-seven patients (66%) had medical comorbidities and 60 patients (52%) had a previous gynecological operation such as a hysterectomy (30.1%), pelvic floor repair (12.9%), and a tape procedure for urinary incontinence (8.6)%. On clinical examination, 71 patients (62%) had evidence of anterior vaginal wall prolapse and 40 patients (34%) complained of associated symptoms such as pelvic pressure (23.2%), incomplete bladder emptying (11.2%), painful micturition (1.7%), fecal urgency, or incontinence (6%).

The most prevalent type of urinary incontinence based on clinical evaluation (prior to urodynamic) was mixed urinary incontinence (Table 1). This was not confirmed on urodynamic evaluation (Table 2).

**Table 1:** Clinical diagnosis prior to UDS USI: urinary stress incontinence; OAB: overactive bladder; UDS: urodynamic investigations

Urinary incontinence symptoms	Number of patients	Percentage
USI	24	21%
OAB	30	26%
Mixed Incontinence	60	52%
Not Mentioned	2	1%
Total	116	100%

**Table 2:** UDS diagnosis DO: detrusor overactivity; USI: urinary stress incontinence; UDS: urodynamic investigations

UDS Diagnosis	Number of Patients	Percentage
DO	28	24%
USI	30	26%
Mixed	29	25%
No DO/USI	28	24%
Not Performed	1	1%

**Figure 1:**
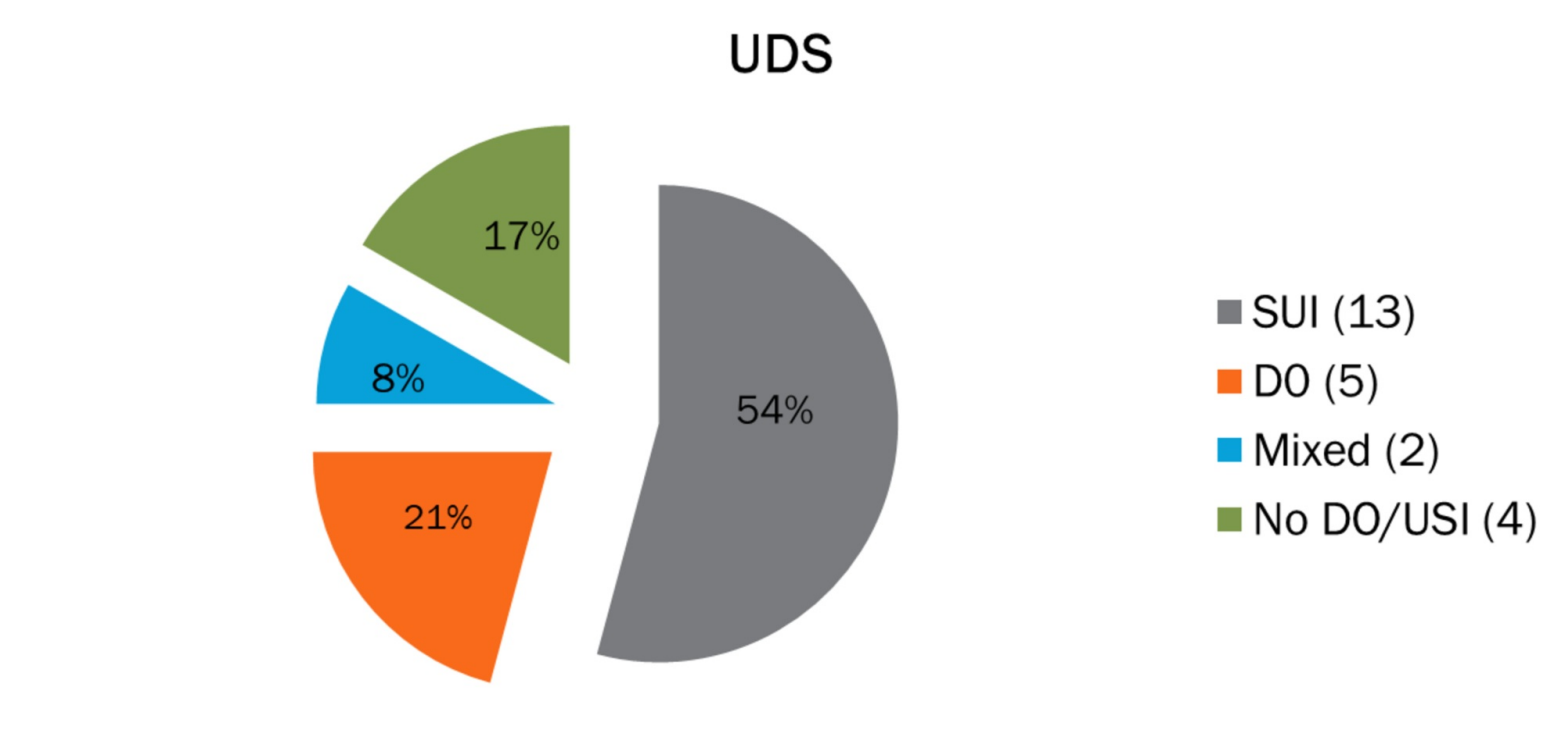
Clinical diagnosis of stress urinary incontinence (N=24) SUI: stress urinary incontinence; DO: detrusor overactivity; Mixed: mixed urinary incontinence

**Figure 2:**
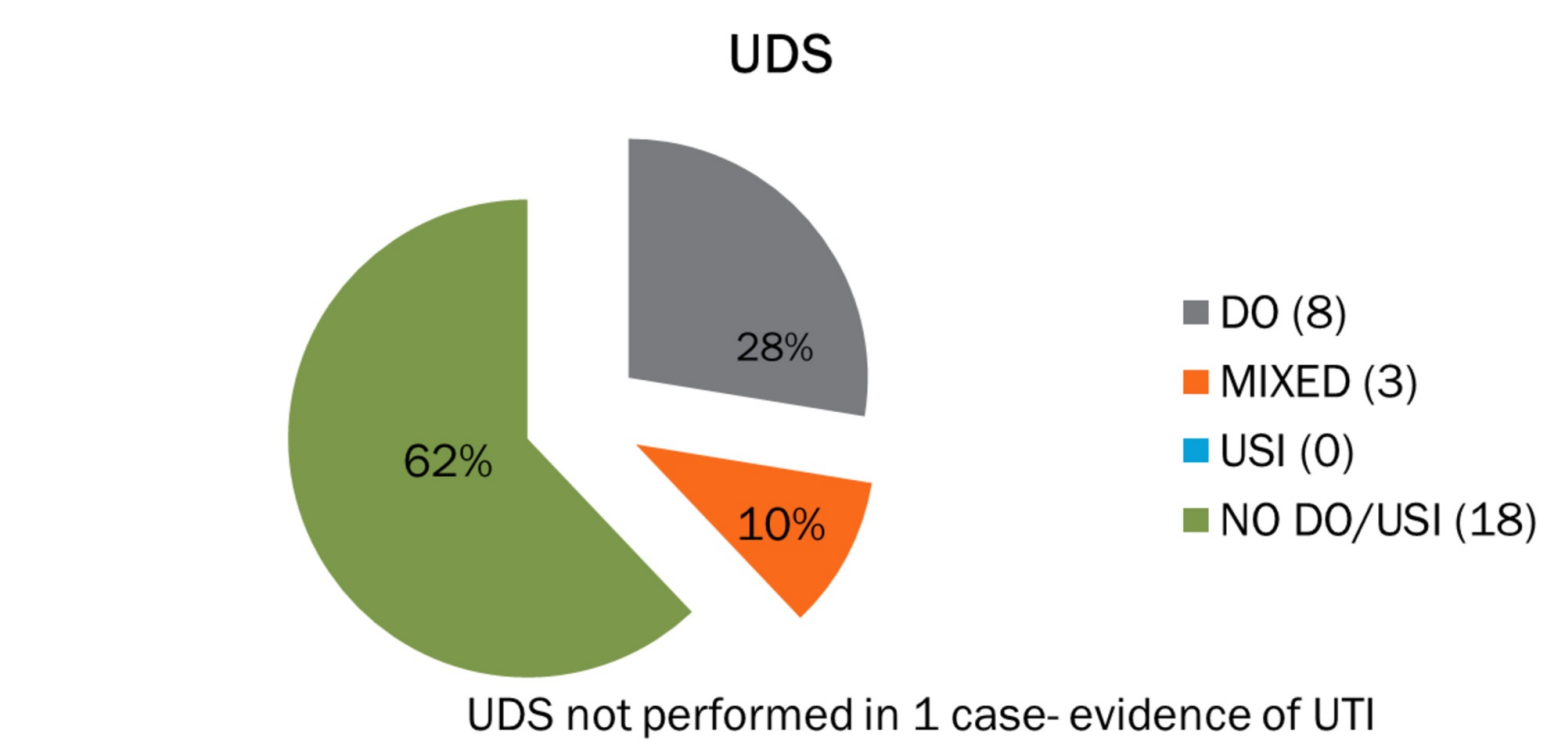
Clinical diagnosis of overactive bladder (N=30) USI: urinary stress incontinence

**Figure 3:**
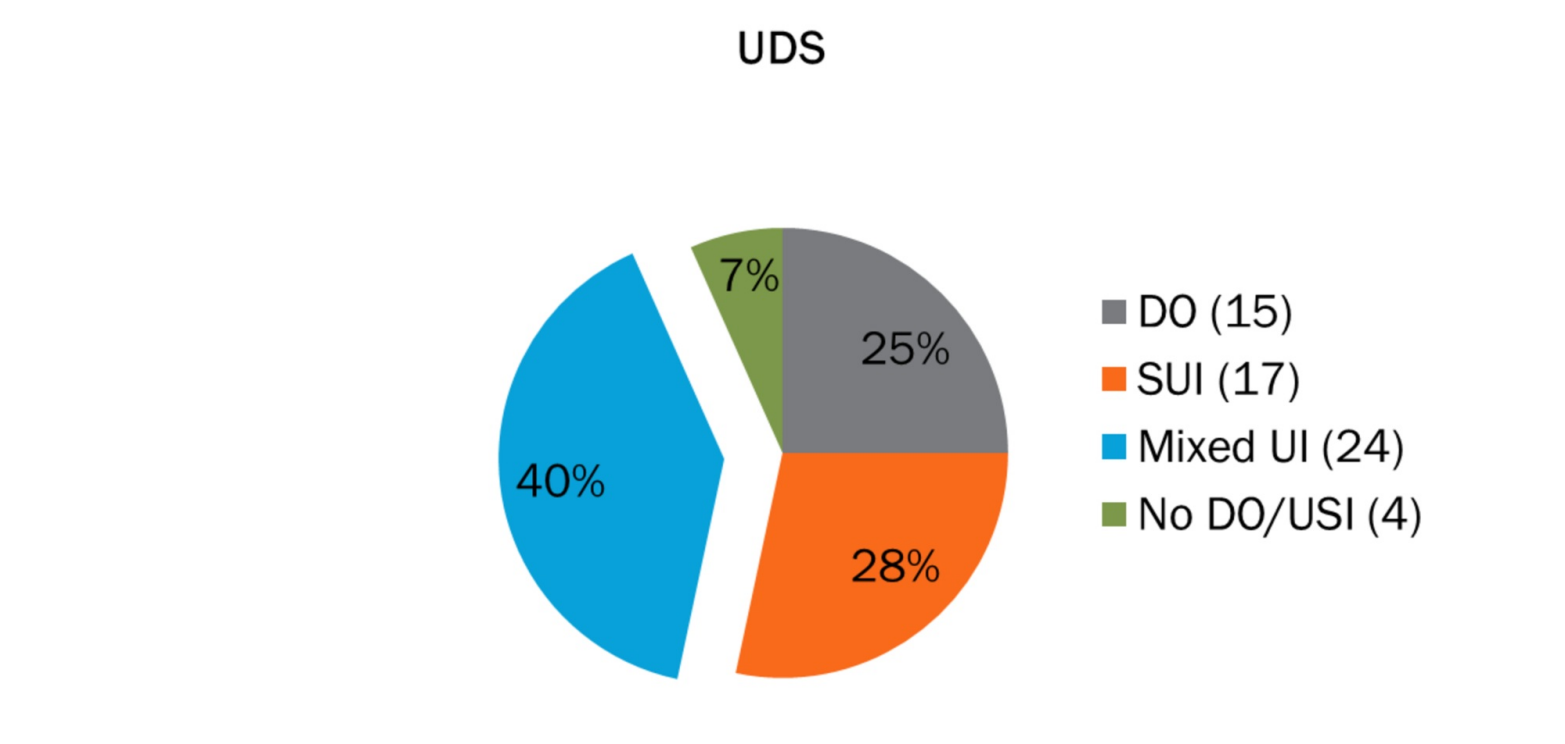
Clinical diagnosis of mixed urinary incontinence (N=60) SUI: stress urinary incontinence; DO: detrusor overactivity; Mixed UI: mixed urinary incontinence

For patients with the clinical diagnosis of stress urinary incontinence, the urodynamic evaluation showed evidence of stress incontinence in 54% of cases, detrusor overactivity in 21%, mixed urinary incontinence in 8%, and no pathology noted in 17% of the patients that were initially referred for urodynamic investigations (Figure 1). For patients with an overactive bladder, the urodynamic evaluation showed no evidence of urinary incontinence in more than half of the cases (62%). Furthermore, in the same group, only eight out of 30 patients (28%) were diagnosed with detrusor overactivity, whereas 10% had mixed urinary incontinence according to the urodynamic evaluation. In one case, urodynamic tests were not performed since there was evidence of urine infection (Figure 2). For patients with clinically suspected mixed urinary incontinence, 40% had both detrusor overactivity and stress incontinence following urodynamics while 28% and 25% had stress incontinence and detrusor overactivity, respectively. The remaining 7% of the group had no pathological urodynamic result (Figure 3).

## Discussion

Urinary incontinence is any uncontrolled leakage of urine, which often has a large impact on the quality of life. The condition can manifest in various ways, with a plethora of symptoms and signs (Table 3). There are three main types of incontinence that we evaluate in this study: stress, urge, and mixed incontinence. Urodynamic investigations are commonly used in the assessment and management of women with urinary incontinence. UDS provides not only objective pathophysiological explanations for symptoms and/or dysfunction of the lower and upper urinary tracts but also information about pre and postvoid flow patterns and consists of a series of invasive tests (Table 4).

**Table 3:** International Continence Society standardized terminology of symptoms, signs and urodynamic observations

Symptoms	
Stress urinary incontinence	Complaint of involuntary loss of urine on effort or exertion, or sneezing or coughing
Urge urinary incontinence	Complaint of involuntary loss of urine accompanied by or immediately preceded by urgency
Mixed urinary incontinence	Complaint of involuntary leakage associated with urgency and with exertion, sneezing or coughing
Signs	
Stress urinary incontinence	Observation of involuntary leakage from the urethra, synchronous with exertion/effort, or sneezing or coughing
Urodynamic observations	
Detrusor overactivity	Urodynamic observation characterized by involuntary detrusor contractions during the filling phase which can be spontaneous or provoked
Urodynamic stress incontinence	Noted during filling cystometry as the involuntary leakage of urine during increased abdominal pressure in the absence of a detrusor contraction

**Table 4:** Urodynamic studies for urinary incontinence assessment

Symptoms	
Stress urinary incontinence	Complaint of involuntary loss of urine on effort or exertion, or sneezing or coughing
Urge urinary incontinence	Complaint of involuntary loss of urine accompanied by or immediately preceded by urgency
Mixed urinary incontinence	Complaint of involuntary leakage associated with urgency and with exertion, sneezing or coughing
Signs	
Stress urinary incontinence	Observation of involuntary leakage from the urethra, synchronous with exertion/effort, or sneezing or coughing
Urodynamic observations	
Detrusor overactivity	Urodynamic observation characterized by involuntary detrusor contractions during the filling phase which can be spontaneous or provoked
Urodynamic stress incontinence	Noted during filling cystometry as the involuntary leakage of urine during increased abdominal pressure in the absence of a detrusor contraction

The present diversity in the success rates after urinary incontinence and bladder neck surgeries denotes the difficulty in arriving at an accurate diagnosis. Furthermore, there is not enough evidence to suggest that UDS use improves clinical outcomes [8]. The NCCWCH 2006 group concluded that: "It has not been shown that carrying out urodynamic investigations before initial treatment improves outcome" [1]. Current literature, including Cochrane and systematic reviews, suggests that there may be a poor correlation between the type of urinary incontinence based on clinical evaluation and the results of urodynamic investigations [6-7,9].

**Figure 4:**
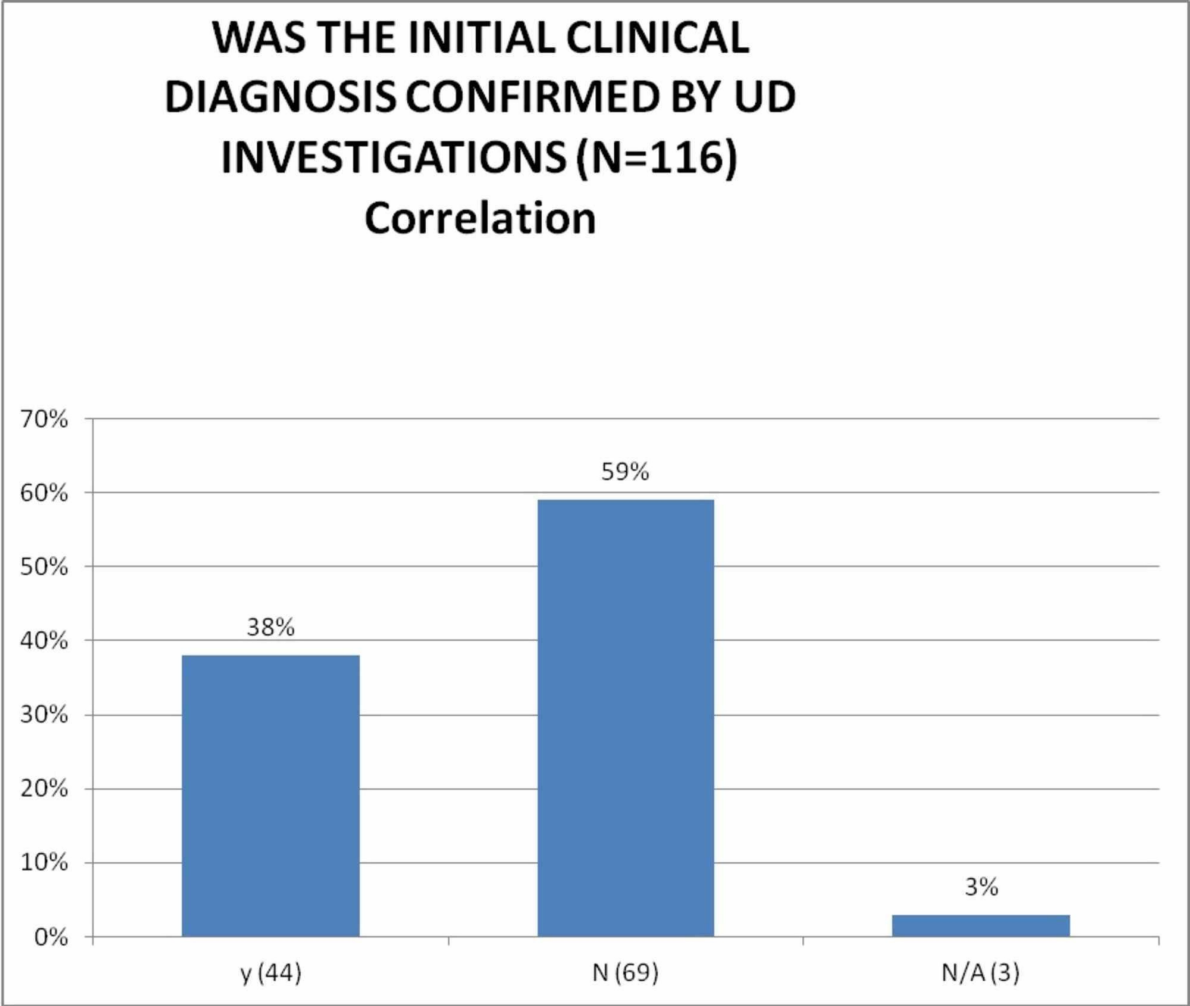
Correlation between clinical and urodynamic diagnoses

In this review, the clinical study of 116 women was summarized in order to investigate the urodynamic tests as a diagnostic tool in the management of urinary incontinence in women. Overall, the clinical diagnosis was confirmed by the results of urodynamic investigations in 38% of patients (Figure 4), which is higher as compared to a previously reported study by McNanley et al. [7]. This study also adds that the reclassification of the type of urinary incontinence was especially high among women with symptoms of an overactive bladder whereas another study suggests that this is more evident for patients with mixed urinary incontinence [6]. The possible clinical implications of that have not been studied in this review.

In addition to this, it is clear that patients referred for stress urinary incontinence were more likely to have this diagnosis confirmed by urodynamic evaluation at 54%. Currently, the Royal College of Obstetricians and Gynaecologists (RCOG) recommends urodynamic testing prior to surgical management for stress incontinence for patients with suspected complicating factors such as voiding dysfunction or detrusor overactivity [4]. There are no meta-analyses or systematic reviews assessing the predictive value of urodynamic preoperatively, yet a randomized controlled trial (RCT) reported that only clinical evaluation prior to stress incontinence surgery was not inferior to urodynamic testing for outcomes one year postoperatively [10]. In combination with the result mentioned above, we find that performing surgery for patients diagnosed with USI by urodynamics should be questioned, and we agree with Hilton et al.'s suggestion that "a definitive trial of invasive urodynamic testing versus clinical assessment prior to surgery for SUI or stress predominant MUI should be undertaken" [11]. The National Institute for Health and Care Excellence (NICE) and the International Consultation on Incontinence for Urodynamic Testing (ICI‐UDT) both recommend urodynamics for overactive bladder symptoms and urge‐predominant mixed urinary incontinence, only after the failure of initial conservative treatment and prior to considering surgery [12-13]. For stress incontinence, NICE recommends urodynamic observations only if there are other factors such as previous surgery for stress incontinence, prolapse, predominant urge mixed urinary incontinence, voiding dysfunction symptoms, or unclear type of incontinence [14].

Taking everything into consideration, the well-known statement of Blaivas in 1996, "Bladder is an unreliable witness," still holds true while urodynamic investigations have a major role in the assessment of lower urinary tract dysfunction in combination with clinical assessments [15]. Rather than being a conclusive diagnosis of the type of incontinence, urodynamic investigations might help in counseling patients about voiding problems that can persist looking at the flow rate, volume, and residual urine. Those who have a large bladder, slow flow rate, and significant postvoid residual volume will need careful review and counseling about the significant risk of intermittent self-catheterization and be prepared prior to any surgical intervention.

## Conclusions

The findings of our study suggest that there is an overall poor correlation between the clinical diagnosis of the type of urinary incontinence and the results of urodynamic investigations. This is in agreement with similar reviews in the current literature. The poor congruity between clinical evaluation and urodynamic testing was more evident in the group of patients with the clinical diagnosis of overactive bladder. The clinician must formulate a clear diagnostic question that they expect the urodynamics to answer. We suggest a careful clinical assessment and the use of tools such as standardized questionnaires to improve the accuracy of the clinical diagnosis of the type of urinary incontinence in women. This study suggests that the effect of urodynamic observations on both the choice of management and the actual outcome after surgical intervention needs further evaluation since it is invasive and time-consuming testing. To conclude, the establishment of the final diagnosis of urinary incontinence and planning for management should be based on a detailed history, physical examination, bladder diaries, and careful interpretation of urodynamic data. The clinician should rely not only on urodynamics but also on the clinical diagnosis and actual symptoms of the patient when considering surgical intervention since urodynamics on its own is not a reliable method and cannot provide a definite diagnosis. Last but not least, clinicians should adhere to the specific indications set by the current guidelines for requesting urodynamic investigations since the amount of information produced by these investigations can be daunting to interpret and challenge the initial diagnosis.
